# Factors associated with contraceptive use among young women in Malawi: analysis of the 2015–16 Malawi demographic and health survey data

**DOI:** 10.1186/s40834-018-0065-x

**Published:** 2018-09-20

**Authors:** Chrispin Mandiwa, Bernadetta Namondwe, Andrew Makwinja, Collins Zamawe

**Affiliations:** 1 Ministry of Health, South–West Zone Health Support Office, P.O. Box 3, Blantyre, Malawi; 2 University of Malawi, Kamuzu College of Nursing, Lilongwe, Malawi; 3 University of Malawi, College of Medicine, Blantyre, Malawi; 4 University College London, Institute for Global Health, London, UK; 5 Malawi Health Sector Programme (DFID Project), Lilongwe, Malawi

**Keywords:** Contraceptive use, Young women, Family planning, Malawi

## Abstract

**Background:**

Although Malawi is one of the countries with highest Contraceptive Prevalence Rate (CPR) in Sub–Saharan Africa, pregnancies and fertility among young women remain high. This suggests low up take of contraceptives by young women. The aim of this study was to investigate the factors associated with contraceptive use among young women in Malawi.

**Methods:**

This is a secondary analysis of household data for 10,422 young women aged 15–24 years collected during the 2015–16 Malawi Demographic and Health Survey (MDHS). The sample was weighted to ensure representativeness. Descriptive statistics, bivariate and multivariate logistic regressions were performed to assess the demographic, social – economic and other factors that influence contraceptive use among young women. Crude Odds Ratio (COR) and Adjusted Odds Ratio (AOR) with their corresponding 95% confidence intervals (95% CI) were computed using the Statistical Package for the Social Sciences version 22.0.

**Results:**

Of the 10,422 young women, 3219 used contraception representing a prevalence of 30.9%. The findings indicate that age, region of residence, marital status, education, religion, work status, a visit to health facility, and knowledge of the ovulatory cycle are significant predictors of contraceptive use among young women in Malawi. Women who were in the age group 20–24 years (AOR = 1.93; 95% CI = 1.73–2.16), working (AOR = 1.26; 95% CI = 1.14–1.39), currently married (AOR = 6.26; 95% CI = 5.46–7.18), knowledgeable about their ovulatory cycle (AOR = 1.75; 95% CI = 1.50–2.05), and those with primary education (AOR = 1.47; 95% CI = 1.18–1.83) were more likely to use contraceptives than their counterparts.

**Conclusion:**

This study has demonstrated that several social demographic and economic factors are associated with contraceptive use among young women in Malawi. These findings should be considered and reflected in public health policies to address issues that could be barriers to the use of contraception by young women. Strengthening access to family planning information and services for young women is highly recommended to reduce pregnancies among young women in Malawi.

## Background

Malawi is among the countries classified by the World Health Organisation (WHO) to have made no progress towards reducing maternal mortality ratio (MMR) between 1990 and 2015 [1]. The country’s MMR is currently estimated to be 439 per 100,000 live births, which is one of the highest in Sub–Saharan Africa (SSS) [2]. Many studies have shown that unintended pregnancies among young women greatly contribute to high maternal and neonatal mortality through increased risk for unsafe abortion, birth injuries and postpartum depression [3–6]. Thus, delaying or avoiding pregnancies among young women is a key intervention in preventing and reducing maternal deaths more especially in countries with high maternal mortality like Malawi.

Family planning (the use of modern contraceptives or traditional methods to limit or space pregnancies) is one of the globally recognized essential strategies for reducing maternal and neonatal mortality, particularly in developing countries where almost all maternal and child mortality occur [7–9]. Family planning reduces mortality risk by preventing (a) unintended pregnancies (thereby reducing maternal deaths caused by unsafe abortion), (b) pregnancy among adolescents (who are at a higher risk of death from childbearing), and (c) closely spaced pregnancies (which improves perinatal outcomes and child survival) [10–12]. Therefore, the role of family planning in reducing maternal morbidity and mortality cannot be overemphasized.

Although Malawi is one of the countries with highest Contraceptive Prevalence Rate (CPR) in Sub–Saharan Africa, pregnancies among young women and fertility remain high [2]. A recent Malawi Demographic Health Survey (MDHS) report indicates that teenage childbearing has increased by 3% between 2010 and 2016, which suggests low use of contraceptives by young women [2]. The determinants of contraceptive use have been explored around the world among women of child bearing age (15–49 years old), but data on the use by young women is limited [13–15]. Understanding the key factors influencing contraceptive use among young women who are at a higher risk of maternal mortality and morbidity could inform interventions to improve uptake of contraceptives among this group. Therefore, the aim of this study was to examine the correlates of contraceptive use among young women in Malawi.

## Methods

### Study design and data source

This is a secondary analysis of cross–sectional household data for women collected during the 2015–16 MDHS. Four questionnaires were used for the data collection: the Household Questionnaire, the Women’s Questionnaire, the Men’s Questionnaire and the Biomarker Questionnaire. The data used in this analysis were collected using the women’s questionnaire.

### Overview of the MDHS: objectives, population and sampling

The MDHS is a nationwide survey with a representative sample of women and men aged 15–49 and 15–54, respectively. It is designed to provide data for monitoring the population and health situation in Malawi [2].

A two-stage cluster sampling procedure was used to generate a nationally representative sample of households. In the first stage, 850 enumeration areas or clusters (173 clusters in urban areas and 677 in rural areas) were selected with probability proportional to sample enumeration area (SEA) size. In the second stage, 30 households per urban cluster and 33 per rural cluster were selected using a systematic random sampling approach. All women of reproductive age (15–49 years) in the selected households were eligible to participate. In the 850 selected clusters, 26,564 households were occupied at the time of data collection of which 26,361 were successfully interviewed, yielding a household response rate of 99%. In total, 24,562 women were successfully interviewed and in this analysis we have included 10,422 young women aged 15–24 years.

### Study variables and measurements

#### Dependent variable

The outcome variable of this study was contraceptive use. Data on contraceptive use was obtained through the women’s questionnaire. Women were asked this question: “Are you or your partner currently doing something or using any method to delay or avoid getting pregnant?” Women who reported current use of either modern or traditional contraceptive methods were considered as current users of contraceptives and those who responded with a ‘no’ were regarded as non-users.

#### Independent variables

The variables were grouped into three categories; socio–demographic variables which included age (in two categories; 15–19 and 20–24), marital status (in three categories; never married, currently married, formerly married), religion (in four categories; catholic, other Christian, Muslim, no religion), region (in three categories; northern, central, south), residence (urban/rural); social-economic variables which included education (in three categories; none, primary school, secondary school or above), wealth index (in three categories; poor, medium, rich) and work status/paid work (working/not working). The other independent factors were participant’s knowledge of ovulatory cycle (yes/no) and woman’s visit to a health facility.

### Ethical consideration

Permission to use the data was obtained from the MEASURE DHS, which is the monitoring and evaluation body of the demographic health survey (DHS) globally. The original study obtained ethical clearance from the Malawi’s National Health Sciences Research Committee (NHSRC). All participants provided oral informed consent**.**

### Data management and analysis

First, we cleaned the data and recoded some of the variables to suit the objective of this study. Descriptive statistics was used to summarize the data and the results were presented as proportions (%). Bivariate analyses (Pearson χ2 square) were conducted to determine the associations between contraceptive use and each of the predictor variables. Variables that had an association with contraceptive use at ≤0.25 on binary logistic regression were further analysed using multivariate logistic regression to identify predictors of contraceptive use [16]. Crude and adjusted odds ratios and their 95% confidence intervals (95% CI) were estimated. To adjust for clustering, all statistical analyses were performed using complex samples analysis of the Statistical Package for the Social Sciences (SPSS, IBM version 22), and statistical significance was set at *P-value* of less than 0.05. All analyses were weighted using a sample weight that was generated for the dataset to account for differences in sampling probabilities.

## Results

### Social demographic characteristics of the study participants

Of the 10,422 (weighted) eligible participants, 3219 used contraception representing a prevalence of 30.9%. Table 1 summarises the characteristics of the study participants. In brief, about half (50.5%) were in the 15–19 years age group while 49.5% were aged 20 to 24 and the majority (81.8%) of the participants were rural dwellers. Nearly half (45.4%) of the participants were from southern region and 46.9% were married. Overall, 18.5% of the participants were Catholics while 12.9% were Muslims and only 0.2% had no religion. Over 60% of the participants had attained primary school education, 49.4% were working and 40.3% were from poor households. Majority (82.7%) of the participants had knowledge of their ovulatory cycle and over half (58.1%) of the participants did not visit any health facility.

**Table 1:** Bivariate association between contraceptive use and various background characteristics

Characteristics			Contraceptive utilization				
	Total		Non-users		Users		*p-value*
	n	%	n	%	n	%	
Age (years)							< 0.001^*^
15–19	5263	50.5	4452	61.8	811	25.2	
20–24	5159	49.5	2751	38.2	2408	74.8	
Residence							< 0.001^*^
Rural	8530	81.8	5833	81.0	2697	83.8	
Urban	1892	18.2	1370	19.0	522	16.2	
Region							0.236^**^
Northern	1159	11.1	818	11.4	341	10.6	
Central	4536	43.5	3099	43.0	1437	44.7	
Southern	4726	45.4	3286	45.6	1440	44.7	
Marital Status							< 0.001^*^
Never married	4828	46.3	4394	61.0	434	13.5	
Currently married	4888	46.9	2387	33.1	2501	77.7	
Formerly married	707	6.8	423	5.9	284	8.8	
Religion							< 0.001^*^
Catholic	1925	18.5	1336	18.5	589	18.3	
Other Christians	7131	68.4	4855	67.4	2276	70.7	
Muslim	1341	12.9	1000	13.9	341	10.6	
No religion	24	0.2	12	0.2	12	0.4	
Wealth							< 0.001^*^
Poor	4200	40.3	2660	36.9	1540	47.9	
Medium	1944	18.7	1339	18.6	605	18.8	
Rich	4276	41.0	3203	44.5	1073	33.3	
Education							< 0.001^*^
None	455	4.4	298	4.1	157	4.9	
Primary school	6739	64.7	4507	62.6	2232	69.4	
Secondary school or above	3227	31.0	2398	33.3	829	25.8	
Work status							< 0.001^*^
Working	5145	49.4	3176	44.1	1969	61.2	
Not working	5277	50.6	4027	55.9	1250	38.8	
Rich							
Visited health facility							< 0.001^*^
Yes	5650	54.2	3328	46.2	2322	72.1	
No	4772	45.8	3875	53.8	897	27.9	
Knowledge of ovulatory cycle							< 0.001^*^
Yes	8619	82.7	5654	78.5	2965	92.1	
No	1803	17.3	1549	21.5	254	7.9	

^*^Significant (*P* < 0.05); ^**^Non-significant (*P* > 0.05)

### Factors associated with contraceptive use

Overall, most of the social and demographic characteristics of participants were significantly associated with contraceptive use (Tables 1 and 2). We found that women in the age group 20–24 years were 93% (AOR = 1.93; 95% CI = 1.73–2.16) more likely to use contraceptives compared to adolescents in the age group 15–19 years. Married (AOR = 6.26; 95% CI = 5.46–7.18) and formerly married (AOR = 3.94; 95% CI = 3.23–4.81) participants had higher odds to use contraceptives than their counterparts who were unmarried. Women who were from central (AOR = 1.22; 95% CI =1.03–1.43]) and southern region (AOR = 1.29; 95% CI = 1.09–1.51) were more likely to use contraceptives than those who were from northern region. In addition, women who had knowledge of their ovulatory cycle had 75% higher odds (AOR = 1.75; 95% CI = 1.50–2.05) to use contraceptives than their counterparts who had no knowledge of their ovulatory cycle. Likewise, women who visited a health facility were 61% (AOR = 1.61; 95% CI = 1.45–1.79) more likely to use contraceptives than their counterparts who had not visited any health facility. Women who attained primary school education were 47% (AOR = 1.47; 95% CI = 1.18–1.83) more likely to use contraceptives than uneducated women. On the other hand, Muslim young women had 49% (AOR = 0.51; 95% CI =0.43–0.61) lesser odds of using contraceptive than Catholic women. Moreover, women who were rural dwellers were 24% (AOR = 0.76; 95% CI = 0.65–0.88) less likely to use contraceptives than those who were urban dwellers.

**Table 2:** Logistic regression of correlates of contraceptive use by young women in Malawi

Characteristics	COR	95%CI	*P-value*	AOR	95%CI	*P-value*
Age (years)						
15–19	Ref.			Ref.		
20–24	4.81	4.38–5.27	< 0.001^*^	1.93	1.73–2.16	< 0.001^*^
Residence						
Urban	Ref.			Ref.		
Rural	1.21	1.09–1.36	< 0.001^*^	0.76	0.65–0.88	< 0.001^*^
Region						
Northern	Ref.			Ref.		
Central	1.11	0.97–1.28	0.141^**^	1.22	1.03–1.43	0.020^*^
Southern	1.05	0.91–1.21	0.496^**^	1.29	1.09–1.51	0.003^*^
Marital Status						
Never married	Ref.			Ref.		
Currently married	10.62	9.48–11.89	< 0.001^*^	6.26	5.46–7.18	< 0.001^*^
Formerly married	6.81	5.69–8.15	< 0.001^*^	3.94	3.23–4.81	< 0.001^*^
Religion						
Catholic	Ref.			Ref.		
Other Christians	1.06	0.95–1.19	0.27^**^	0.87	0.77–0.99	0.034^*^
Muslim	0.77	0.66–0.91	< 0.001^*^	0.51	0.43–0.61	< 0.001^*^
No religion	2.40	1.07–5.37	0.033^*^	1.66	0.64–4.30	0.278^**^
Wealth						
Poor	Ref.			Ref.		
Medium	0.78	0.70–0.88	< 0.001^*^	0.99	0.87–1.13	0.930^**^
Rich	0.58	0.53–0.64	< 0.001^*^	0.93	0.82–1.06	0.278^**^
Education						
None	Ref.			Ref.		
Primary school	0.94	0.77–1.15	0.542^**^	1.47	1.18–1.83	< 0.001^*^
Secondary school or above	0.66	0.53–0.81	< 0.001^*^	1.24	0.97–1.57	0.083^**^
Work status						
Not working	Ref.			Ref.		
Working	2.00	1.84–2.17	< 0.001^*^	1.26	1.14–1.39	< 0.001^*^
Visited health facility						
No	Ref.			Ref.		
Yes	3.02	2.76–3.30	< 0.001^*^	1.61	1.45–1.79	< 0.001^*^
Knowledge of ovulatory cycle						
No	Ref.			Ref.		
Yes	3.20	2.78–3.68	< 0.001^*^	1.75	1.50–2.05	< 0.001^*^

*AOR* adjusted odds ratio, *COR* crude odds ratio, *Ref* reference category

^*^Significant (*P* < 0.05); ^**^Non-significant (*P* > 0.05)

**Fig. 1:**
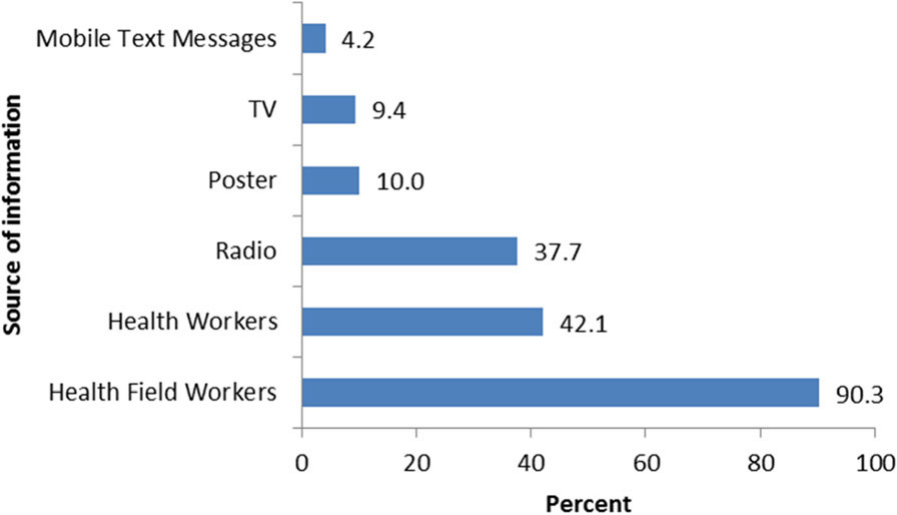
Distribution of sources of contraceptive methods information for young women in Malawi

The distribution of sources of information for contraceptive methods for young women was assessed graphically. Majority of the participants (90.3%) heard about contraceptive methods from health field workers while only 4.2% got information for contraceptive methods from mobile text messages as shown in Fig. 1.

## Discussion

The findings indicate that most of the social, economic and demographic characteristics are significant predictors of contraceptive use among adolescents and young women in Malawi. We observed that women in the age bracket of 20–24 years were more likely to use contraceptives than their counterparts aged 15–19 years. This observation could partly explain the rise in Malawi’s teenage childbearing from 26% in 2010 to 29% in 2016 [2] . It is assumed that women aged 20–24 years understand the consequences of engaging in unprotected sexual act or without contraceptive use compared to adolescents. Additionally, most of women aged 15–19 years might be newly married, and they may take marriage as an institution of producing children. Adolescents may also have problems in accessing FP services because they may not know where to obtain contraception or cannot afford services. Our results concurs with the findings of previous studies in Ethiopia, Nepal and Uganda which also reported reduced contraceptive use among adolescents compared to women aged 20–24 years [17–19] .

The results further showed disparities in contraceptive use among women by region (southern, central and northern) and residence (Urban vs. Rural). For example, women in central and southern region were more likely to use contraceptives than those in northern region. Similarly, women in rural areas were less likely to use contraceptives than women in urban areas. This is in agreement with results from studies conducted in other countries, which reported that women living in urban area were more likely to use contraceptives and experience delayed age at marriage than women living in rural area [20–22]. Differences in cultural beliefs and values between rural and urban as well as across regions are some of the possible explanations for the observed disparities in contraceptive use. Moreover, limited availability of health facilities in rural area might further limit access to and use of contraceptives [23]. Thus, program implementers should consider these issues when designing contraceptive programmes for young women. Besides, this highlights the need for further qualitative studies to investigate the actual reasons for these observed variations.

The findings also show that women who had attained primary school education had higher odds of using contraceptives than their uneducated counterparts. This finding is consistent with previous studies that have shown a similar pattern of relationship between educational status and contraceptive use [24–28]. Education empowers women to have autonomy in making important decisions regarding fertility related issues and also help them exercise reproductive health rights compared to uneducated women. Moreover, educated women could probably have a better understanding of the benefits of using contraception to reduce unintended pregnancies than women with no education. For that reason, it is necessary that family planning service providers must place special emphasis on, and address the needs of women with no or little education during family planning sessions to provide them with basic reproductive health knowledge to improve uptake of contraceptives. Moreover, it is important for policy makers in Malawi to formulate and enforce policies that promote education of girls and women.

We further observed that work status was a significant predictor of contraceptive use among participants as women who were working were more likely to use contraceptives than women who were not working. The possible explanation for this relationship is that women who are working are too preoccupied with work related activities to having babies as a result they may use contraceptives. Additionally, women who are working are likely to be educated, exposed to contraceptive information and may be able to afford contraceptives than those who are not working. Therefore, there is a need for collaborated efforts by government and its partners to make contraceptives affordable to all women. Our finding is consistent with studies done in other countries which also reported that women who were working were more likely to use contraceptive [29–31].

It was also noted that knowledge of ovulatory cycle had a positive significant relationship with use of contraceptives by young women in Malawi. It is possible that women who know their ovulatory cycle may use contraception methods to protect themselves from getting pregnancy during their ovulation period than their counterparts who do not know their ovulatory cycle. This finding is in agreement with results from a study in Ghana, which reported that women who knew their ovulatory cycle were likely to use contraceptives compared to those who did not know their cycle [29].

We observed a positive significant association between a visit to a health facility and contraceptive use. Perhaps, women who visit health facilities have access or are exposed to sexual and reproductive health services than those who do not visit such facilities. Besides, women who want to or are using contraceptives may also likely visit a health facility. This finding concurs with a recent study in Ethiopia which has reported that women who visited a health facility had 54% higher odds of using contraceptives [32]. The results of the present study also indicate that majority of young women heard about contraceptive methods from health field workers. This finding is consistent with a study in Bangladesh which reported that many young women mentioned health field workers as a primary source of information for contraceptive methods. Provision of adequate and correct information on contraception to women can positively impact the utilisation of contraceptives and may reduce unintended pregnancy [33]. Thus, a focus on field health workers through outreach visits can improve uptake of contraceptives among young women.

Even though other studies have suggested that contraceptive use is associated with wealth index, [34–36] our study did not. These conflicting results could possibly be due to different sample size, study participants and setting.

The findings of this study should be considered in light of the following limitations and strengths. First, we used secondary data and some important independent variables of contraceptive use were not available. Second, DHS is cross-sectional in nature as such we cannot establish temporal linkages. Third, contraceptive use was based on self-reported. So, recall bias cannot be ruled out. Fourth, this study did not differentiate the types of contraception (modern and traditional) during analysis. Independent variables may have different influence on the type of contraception. Nevertheless, the main interest in our study was identifying the determinants of contraceptive use among young women in general, not on a specific type of contraception. Future studies need to be conducted to assess if the variables have different or similar effects on modern versus traditional contraceptive methods. The strengths of this study include the use of nationally representative data with relatively large sample size, which imply robust statistical significance.

## Conclusion

The findings of this study highlight the influence of age, type of residence, region of residence, marital status, education, religion, a visit to health facility, work status and knowledge of the ovulatory cycle as key predictors of contraceptive use among young women in Malawi. These findings should be considered and reflected in public health policies to address issues that could be barriers to the use of contraception by young women. Improving access and use of contraception is highly recommended to reduce teenage pregnancies in Malawi.

## Abbreviations

CPR: Contraceptive prevalence rate
MDHS: Malawi demographic health survey
MMR: Maternal mortality ratio
NSO: National statistical office
SSS: Sub–Saharan Africa
STI: Sexually transmitted infections
WHO: World Health Organisation
